# A Case of Adalimumab-Induced Granulomatous Interstitial Nephritis

**DOI:** 10.7759/cureus.15986

**Published:** 2021-06-28

**Authors:** Rory Plant, Adeel Rafi Ahmed, Teresa Mchale, Louise Giblin

**Affiliations:** 1 Nephrology, University Hospital Galway, Galway, IRL; 2 Health Sciences, University of South Wales, Newport, GBR; 3 Pathology and Laboratory Medicine, University Hospital Galway, Galway, IRL

**Keywords:** adalimumab, granulomatous interstitial nephritis, tumour necrosis factor-α (tnf-α), : acute kidney injury, chronic kidney disease (ckd), drug reaction, anti-tumour necrosis factor-alpha (anti-tnf-alpha)

## Abstract

Adalimumab is a monoclonal antibody targeting tumour necrosis factor-alpha (TNF-alpha) and is used for the treatment of numerous autoimmune conditions. There is a paucity of evidence linking adalimumab with granulomatous interstitial nephritis (GIN). We describe a renal biopsy-proven case of GIN secondary to adalimumab therapy. A 52-year-old gentleman with a background of psoriatic arthropathy was referred to the nephrology department by his general practitioner with a progressive decline in renal function over 18 months after initiating adalimumab. A renal biopsy confirmed tubulointerstitial nephritis with focal aggregates of histiocytes, organized as granulomata. Screening for other GIN causing aetiology, including tuberculosis (TB) and sarcoidosis, was negative. Adalimumab was withheld, leading to a slow improvement in renal function over a course of six months. It is essential to monitor renal function when administrating anti-TNF alpha agents as they can rarely paradoxically cause autoimmune reactions such as GIN seen in our case.

## Introduction

Acute interstitial nephritis (AIN) is an important cause of acute kidney injury (AKI) characterized by an inflammatory infiltrate in the renal interstitium, most commonly secondary to a drug reaction [1]. A histological variant of AIN is granulomatous interstitial nephritis (GIN), which is seen in less than 1% of native renal biopsies [2]. Common aetiologies associated with GIN are sarcoidosis, infections (particularly with *Mycobacterium tuberculosis*) and antibiotics [1]. Adalimumab is a monoclonal antibody targeting tumour necrosis factor-alpha (TNF-alpha) and is used to treat autoimmune conditions such as rheumatoid arthritis, psoriatic arthritis, and inflammatory bowel disease. Rarely as a side effect, anti-TNF alpha agents can cause sarcoid-like granulomatosis [3]. However, adalimumab-associated GIN is an under-reported entity. We describe a case of GIN in a patient with psoriasis being treated with adalimumab.

## Case presentation

A 52-year-old gentleman with a background of psoriatic arthropathy was referred to the nephrology department by his general practitioner (GP) after noticing a doubling of serum creatinine from a baseline of 92 μmol/L (1 mg/dL) to 205 μmol/L (2.3 mg/dL) with new-onset hypertension on yearly review. A 24-hour ambulatory blood pressure (BP) monitor showed a mean BP of 152/98 mm/Hg. On further review, his last normal baseline creatinine of 92 μmol/L (1 mg/dL) was 11 months previously. Six months before his current visit, his creatinine done at another service was 108 μmol/L (1.2 mg/dL). He was advised to increase his fluid intake with no other significant changes in management.

His only medication was adalimumab that he had been taking every two weeks for the last 18 months. He had previously been taking methotrexate 20 mg, once weekly, for the last seven years. However, on introducing adalimumab, his dose was tapered down. He was on 5 mg of methotrexate at the time of the elevated serum creatinine reading but had stopped two weeks prior to referral. He denied ever taking non-steroidal anti-inflammatory drugs (NSAIDs) or ever taking proton pump inhibitors (PPI).

On examination, his psoriasis was well controlled. There was no joint pain, weight loss, night sweats, fever, sinusitis, or haemoptysis. A urine dipstick demonstrated +1 protein with a trace of blood. He was commenced on lercanidipine 20 mg to control his BP.

Investigation

Chest x-ray and a renal ultrasound revealed no significant abnormality. Previous investigations prior to adalimumab induction were negative for any infections. Laboratory Investigations revealed a weakly positive anti-nuclear antibody (ANA) 1:80 speckled pattern. Anti-neutrophil cytoplasmic antibodies (ANCA) and anti-glomerular basement membrane (anti-GBM) antibodies were negative. Complement levels were normal.

Serum protein electrophoresis (SPEP) did not demonstrate any monoclonal band, and the serum-free light chain (SFLC) ratio was normal (1.50). Adjusted calcium level was mildly elevated at 2.66 mmol/L (2.2-2.6 mmol/L). The rest of the biochemistry and the haematological screen were normal (Table 1).

**Table 1:** Laboratory investigations

Lab Investigations	Result
Sodium (135-145 mmol/L)	140
Potassium (3.5-5.2 mmol/L)	5
Serum-corrected calcium (2.2-2.6 mmol/L)	2.66
Haemoglobin (13-17 g/dL)	14
White cell count (4.5 to 11.0 × 10^9^/L)	6
Platelets (150,000 to 450,000)	300,000
Complement levels	Normal
Anti-neutrophil cytoplasmic antibodies (ANCA)	Not detected
Anti-nuclear antibodies (ANA)	1:80 speckled pattern
Anti-glomerular basement membrane antibodies (anti-GBM)	Not detected
Tuberculosis/HIV/hepatitis B and hepatitis C polymerase chain reaction (PCR)	Not detected
Serum protein electrophoresis (SPEP)	No monoclonal band
Serum-free light chain (SFLC) ratio (0.26–1.65)	1.50

**Figure 1:**
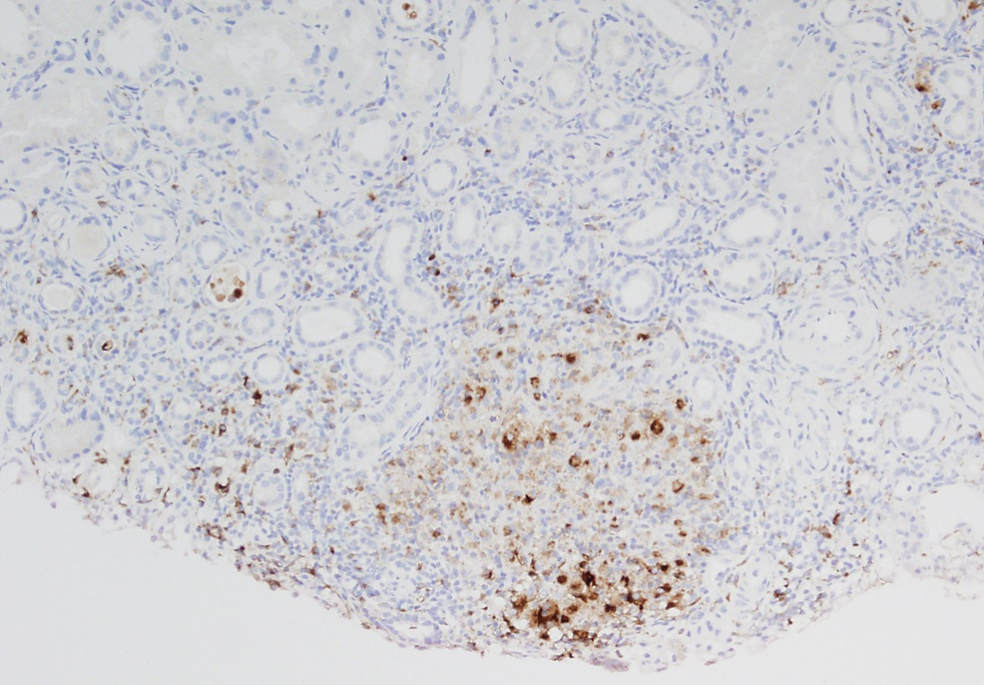
CD68 immunohistochemical stain (a histiocyte marker) highlighting the granuloma

**Figure 2:**
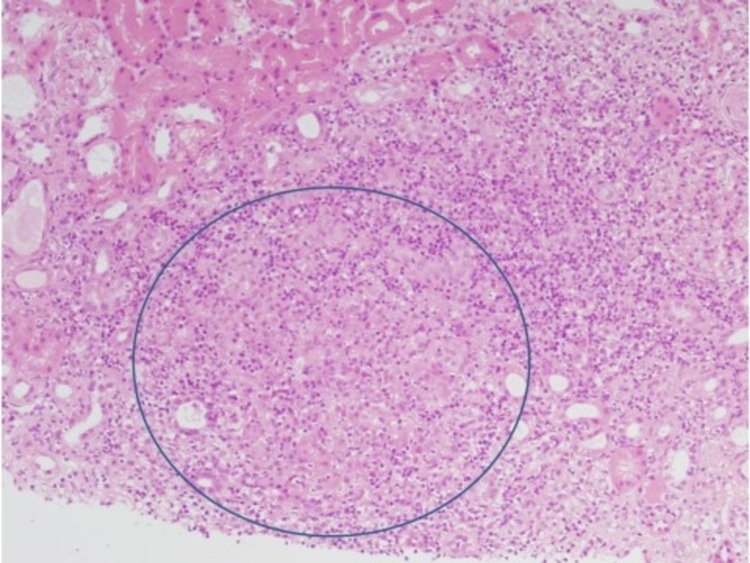
High power (10x): an area of epithelioid histiocytes (circled) forming a granuloma

A renal biopsy was performed. The biopsy showed a chronic patchy tubulointerstitial nephritis with focal aggregates of histiocytes (confirmed by CD68 staining) (Figures 1, 2). These were arranged as loosely formed granulomata. Stains for acid-fast and fungal organisms (Ziel Nielson and Grocott) were negative. The glomerular compartments were largely preserved (one sclerotic glomerulus in 80 sampled). There was evidence of mild tubular injury. Immunofluorescent microscopy using standard antisera panels were negative.

Treatment

The consensus opinion between nephrology and the renal pathologist was that the patient should stop adalimumab therapy and reassess his renal function in six weeks. The use of corticosteroids was discussed; however, it was decided to use it only if there was no improvement in serum creatinine post-withdrawal of adalimumab due to a significant side-effect profile. The case was discussed with dermatology and rheumatology, and it was decided to commence ustekinumab, a human monoclonal antibody that binds to and interferes with interleukin (IL)-12 and IL-23 as an alternate to adalimumab for psoriatic arthropathy once the renal function had stabilized.

Outcome

**Figure 3:**
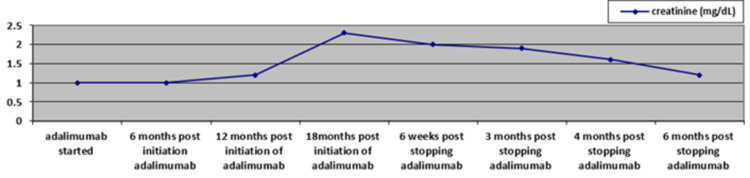
Creatinine trend in relation to adalimumab

Renal function improved after a six-week duration with serum creatinine at 180 μmol/L (2 mg/dL) from a peak of 205 μmol/L (2.3 mg/dL), further supporting that the patient had suffered adalimumab associated GIN. A further 12 weeks later, his renal function was performed and showed serum creatinine of 170 μmol/L (1.9 mg/dL), and a further six weeks later, it reduced to 142 μmol/L (1.6 mg/dL). In his final visit to the nephrology services six weeks later (six months post stopping adalimumab), the creatinine was 110 μmol/L (1.2 mg/dL) (Figure 3). His GP followed him after that.

## Discussion

GIN is a rare histological diagnosis mostly seen secondary to sarcoidosis, TB, and antibiotics [1]. It is present in between 0.5% and 0.9% of native renal biopsies [2]. There is a growing link between anti-TNF-alpha agents and de novo granulomatous disease [4,5]. Our case follows a temporal pattern between the initiation of adalimumab and significant kidney dysfunction at 18 months. Similar to previous cases, withdrawal of the drug led to subsequent stabilization of serum creatinine. Previous cases have also used corticosteroids concomitantly with the withdrawal of the offending agent to reduce the risk of renal injury [5]. The timing of onset of GIN post introduction of adalimumab is variable but available data suggest around 10-12 months [5]. Some residual renal damage may remain highlighted by persistently elevated creatinine compared to baseline, as seen in our case.

The exact mechanism of adalimumab-induced GIN is unknown. Both anti-TNF-alpha therapies and interferon (IFN)-γ have been reported to cause ANA production, overt systemic lupus erythematosus and sarcoidosis affecting the lungs and lymph nodes [3,6]. It has been suggested that anti-TNF-alpha therapies may restore a Type 1 T-helper cells (Th1) response leading to induction of IFN-γ, subsequently precipitating granulomatosis [3].

However, anti-TNF-alpha agents have also been used as second-line treatment in managing steroid-resistant sarcoidosis. Few case reports have successfully used them to treat sarcoid-associated GIN, implicating a more complex immunological phenomenon that needs more extensive controlled studies [7].

Renal function should be monitored while on anti-TNF-alpha therapy. It is recommended that individuals on adalimumab should be monitored for systemic signs of disease such as mouth ulcers, chills, bruising, pallor, and signs of autoimmunity [8]. They further add that individuals should have had a full blood count, liver function test and urea, creatinine, and electrolytes monitored monthly for the first three months and then three monthly thereafter [8]. Observational data have demonstrated the development of autoantibodies, particularly ANA and to a lesser extent anti-double-stranded DNA (anti-dsDNA) antibodies in patients managed with anti-TNF-alpha therapy [9]. Thus, some authors suggest checking for baseline ANA and anti-dsDNA levels when initiating anti-TNF-alpha therapy [9].

Of note, the monitoring of BP is not explicit in the monitoring guidelines, unlike other immunosuppressant drugs such as ciclosporin [8]. Although we are reporting a rare side-effect of anti-TNF-alpha therapies, our case is the second case that illustrates that a rise in BP was central to identifying renal injury [5]. This highlights the importance of routine BP monitoring for those taking biological agents. An explicit reminder of BP monitoring may promote earlier identification of GIN and prevent further under-reporting of this phenomenon. We would further support a low threshold for a kidney biopsy in patients with an asymptomatic decline in renal function who are taking TNF-alpha inhibitors.

## Conclusions

Adalimumab-associated GIN is rarely described in the literature; however, it can cause significant kidney injury if not promptly identified and managed. It is pertinent for clinicians to have a high diagnostic suspicion in patients on anti-TNF alpha agents with deteriorating renal function, new-onset hypertension, and possibly urine dipstick positive for blood and protein. Despite being used to manage a variety of autoimmune conditions, adalimumab can paradoxically induce immune-mediated damage in the kidneys. This may be a class effect as other anti-TNF alpha therapies have been implicated in causing sarcoidosis and lupus. Thus, it is critical to monitor patients using clinical and lab-based parameters. The management involves stopping adalimumab and, on occasions using corticosteroids to reduce interstitial inflammation.
